# Accuracy of Motion Trajectory Achieved with an Intraoral Scanner: An In Vitro Study with a Proof-of-Concept

**DOI:** 10.3390/diagnostics14232713

**Published:** 2024-12-01

**Authors:** Hwa Jung Lee, Kyung Chul Oh

**Affiliations:** Department of Prosthodontics, Yonsei University College of Dentistry, 50-1 Yonsei-ro, Seodaemun-gu, Seoul 03722, Republic of Korea; hjlee0227@yuhs.ac

**Keywords:** motion trajectory, intraoral scanner, jaw-tracking, articulator

## Abstract

**Background:** With the advancement of digital technology, it has become possible to record jaw motion using intraoral scanners. However, there is a paucity of studies evaluating their accuracy. **Methods:** Twelve sets of scan data from 12 individuals were additively manufactured using a 3D printer and 3D-printable material. Each pair of scan data was mounted onto a semi-adjustable articulator. A blue articulating paper was inserted between the mounted models, and the pin of the articulator was moved to simulate motion (ART group). Subsequently, intraoral scan data were obtained, and the movements of the articulator were recorded. The trajectory expressed in the intraoral scanner software (TRIOS3; 3Shape A/S, Copenhagen, Denmark) appeared red on the monitor screen (IOS group). The blue and red areas in the ART and IOS groups, respectively, were measured in pixels for each tooth type, and the number of trajectories marked or expressed for each tooth type was counted. **Results:** Regarding the areas of trajectory, significant differences were observed between the ART and IOS groups across all tooth types. Statistically significant differences were also noted in the number of trajectories for the first premolars and first molars between the two groups. **Conclusions:** Intraoral scanners may not accurately reproduce motion movements at the current level of technology. However, these results should be interpreted with caution because defining the trajectory accuracy between the two groups is challenging unless the (two) trajectories are exactly the same.

## 1. Introduction

Teeth can be lost due to various causes, such as dental caries, periodontal diseases, and trauma. It is crucial to restore them prosthetically in a timely manner. When a new prosthesis is placed in the mouth, it is important to not only achieve static maximal intercuspation but also achieve a harmonious relationship with the opposing teeth during lateral and protrusive movements to avoid disturbance during masticatory behavior and natural jaw movements [1,2]. Therefore, an accurate recording of the mandibular movement trajectory is essential in prosthodontics, as it can minimize unwanted adjustment of the prostheses both at the dental laboratory and chairside in clinics [3]. Precise documentation and pre-analysis of mandibular anterior and lateral movements are pivotal in reducing occlusal interferences during the prosthesis fabrication process. Such precision enables dental technicians to design prostheses that accurately reflect the patient’s functional occlusal dynamics, preventing maladaptive or uncomfortable occlusal interferences in the oral cavity. Additionally, this approach significantly reduces the time required for occlusal adjustments, thereby shortening clinical chair time and improving efficiency for the clinician.

Various attempts have been made to reproduce mandibular movement in individuals [4,5,6,7]. A mechanical articulator is employed to simulate the functional impact of dysmorphology and malocclusion. However, such mechanical devices are limited in their capacity to accurately replicate the variability of biological systems, particularly the dynamic conditions of jaw movements and related factors. Additionally, dental casts fail to fully represent the real-time dynamic occlusal conditions present in the oral cavity. Furthermore, various issues related to technical procedures and dental materials further reduce the precision of these reproductions [6]. Although a semi-adjustable articulator is such a device, it is impossible to fully reproduce the original path of mandibular movements because of its relatively simple working principle, which connects only the first and last movement points. Conversely, a fully adjustable articulator aims to reproduce the real mandibular movements that occur in each individual [8]. However, it is still impossible to reproduce the exact complicated human jaw movements using these fully adjustable articulators. Additionally, a significant drawback of this device is that it is bulky and uncomfortable. 

With the advancement of digital technology, several jaw-tracking devices have been proposed [5,9,10]. In a study on dynamic jaw tracking, a digital approach utilizing a target tracking system and a structured-light three-dimensional scanner was proposed to enhance the accuracy and efficiency in capturing jaw movements [9]. Another study proposed a jaw motion tracking method utilizing a readily accessible home digital camcorder. The findings demonstrated that this method could achieve accuracy comparable to that of conventional jaw-tracking systems [10]. A technique for registering and reproducing individual mandibular movements using an optoelectronic jaw movement analyzer combined with a specialized robotic system was introduced [5]. This approach allowed for the precise tracking and replication of jaw dynamics, contributing to improved accuracy in dental procedures. Physical markers have the advantage of recording mandibular movements with high accuracy and capturing complex motion patterns. However, the requirement of attaching large devices to the face may cause patient discomfort and potentially interfere with natural mandibular motion [11]. Additionally, the setup and operation of such equipment can be intricate and time-consuming. Recently, intraoral scanners have emerged as a viable alternative for recording mandibular movements [12]. These scanners eliminate the need for external facial attachments, enabling the comfortable and unobstructed documentation of natural mandibular motion. Intraoral scanners also offer several advantages in dentistry, including improved accuracy, reduced patient discomfort, and the elimination of traditional impression materials. Moreover, their compact design, compared to large conventional equipment, facilitates seamless integration of scanned data into computer-aided design/computer-aided manufacturing (CAD/CAM) software for prosthetic design and the correction of occlusal discrepancies.

However, the limitations include high initial costs, potential challenges in maintaining the scanning accuracy in certain clinical situations, and the need for continuous technological updates to ensure optimal performance. Additionally, the current digital workflow for occlusal design predominantly relies on the principles of static occlusion [13]. The Patient Specific Motion (PSM; 3Shape A/S, Copenhagen, Denmark) is a markerless mandibular motion tracking system integrated within an intraoral scanner. PSM captures dynamic occlusal records during eccentric mandibular movements by directly accumulating motion data within the patient’s oral cavity. This enables the visualization of mandibular movements and facilitates the correction of occlusal discrepancies within CAD software. By reproducing functional mandibular movements in the CAD environment, PSM may effectively minimize occlusal interferences during eccentric movements [14]. However, few studies have evaluated the accuracy of PSM in recording mandibular movements, highlighting the need for further research in this area. The present study aimed to compare the trajectory expressed through motion scanning in vitro using an intraoral scanner with the actual path obtained using articulated sets of models. The null hypothesis was that the trajectory expressed by motion scanning using an intraoral scanner would not differ from the actual trajectory in an in vitro environment.

## 2. Materials and Methods

Twelve sets of scan data from 12 individuals were additively manufactured using a 3D printer (NextDent5100; NextDent B.V., Soesterberg, The Netherlands) and a 3D-printable material (NextDent Model 2.0; NextDent B.V., Soesterberg, The Netherlands), with a build orientation angle of 45°. Each pair of 3D-printed models was mounted onto a semi-adjustable articulator (PROTARevo 7; KaVo, Biberach, Germany). A blue articulating paper (BK11; Bausch, Nashua, NH, USA) was inserted between the mounted models on the right quadrant, and the pin of the articulator was moved freely to simulate motion. Thereafter, the trajectory in the mounted models was shown in blue. Subsequently, the same quadrants were scanned, the inter-arch relationship was obtained, and the movements of the articulator reproduced by moving its pin were recorded using an intraoral scanner (TRIOS3; 3Shape A/S, Copenhagen, Denmark). The trajectory expressed in the software embedded in the intraoral scanner appeared red on the monitor screen.

**Figure 1:**
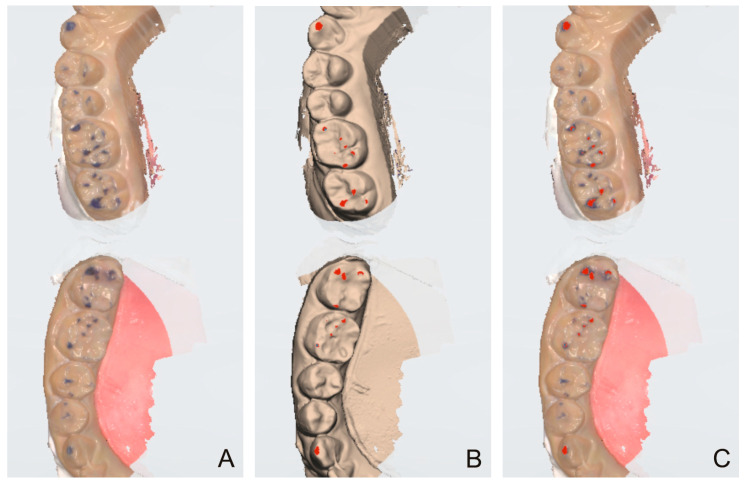
Representative images of the screen capture of the trajectories. (**A**) The actual trajectory marked with the blue articulating paper. (**B**) The trajectory in red color exhibited by the intraoral scanner. (**C**) A superimposed image exhibiting the trajectories in both groups.

**Figure 2:**
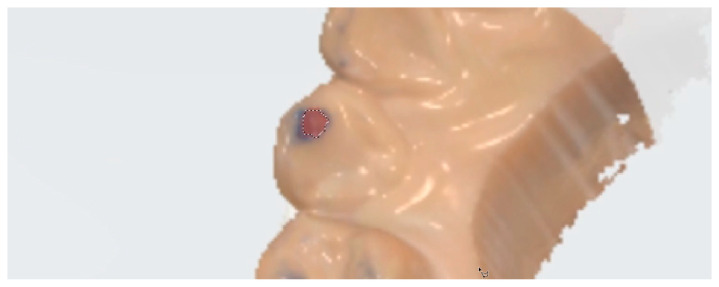
Representative image illustrating the measurement procedures for the blue and red areas using an im-age-editing software (Photoshop). The dotted line outlines the areas to be measured for the maxillary right canine.

Because the 3D coordinates of the models should not be changed for comparison, a screen capture of the monitor interface was performed. First, the texture mode was turned off so that colors were visible, including the trajectory expressed with the blue articulating paper, and the status was captured (ART Group) (Figure 1A). Subsequently, the texture mode was turned on, and the recording of articulator movements was activated so that the red-colored trajectory exhibited by the intraoral scanner was visible (IOS Group) (Figure 1B). The captured images were imported into an image-editing software (Photoshop) and superimposed onto each other (Figure 1C). Subsequently, the blue and red areas in the ART and IOS groups were measured in pixels (Figure 2). These areas were subdivided into tooth type-based levels for further analysis. Additionally, the number of trajectories marked (i.e., ART group) or expressed (i.e., IOS group) for each tooth type was counted.

The Wilcoxon matched-pairs signed-rank test was performed using statistical software (IBM SPSS Statistics for Windows, Version 25.0; IBM Corp., Armonk, NY, USA) to comparatively evaluate the areas of the trajectory and the differences in the number of trajectories between the two groups based on each tooth type analysis. The level of statistical significance was set at α = 0.05.

## 3. Results

Regarding the areas of trajectory, the two groups had significant differences in all tooth types (canine, *p* = 0.002; first and second premolars and molars, *p* < 0.001; Table 1). Regardless of the tooth types, the areas of trajectories were significantly larger in the ART group compared to the IOS group. The number of trajectories was significantly higher in the ART group compared to the IOS group in the first premolar and first molar (*p* = 0.005 and *p* = 0.007, respectively), whereas no significant differences were observed in the canine, second premolar, and second molar (*p* = 0.240, *p* = 0.181, and *p* = 0.616, respectively) trajectories.

## 4. Discussion

The null hypothesis was rejected because there were significant differences between the two groups in terms of the area and number of trajectories on the first premolar and molar. The wider contact areas recorded in the ART group may be attributed to the inherent thickness of the articulating paper, which allows it to register even slight brushing contacts. In contrast, the PSM feature of the intraoral scanner calculates and displays trajectories based on its internal algorithms. Consequently, regions where the articulating paper registered contact because of its thickness may have been interpreted as non-contact areas by the scanner. Despite these differences, the overall trends in the results were similar between the two groups. This suggests that the PSM approach, incorporating mandibular movements, could offer significant advantages for prosthesis fabrication as the technology continues to evolve.

Precisely fabricated fixed dental prostheses show a good occlusal relationship in static circumstances in the maximum intercuspation state and a harmonious occlusal scheme during dynamic movement [3]. In other words, the occlusal adjustment time can be reduced, and the original and intended esthetic shape and form of the prostheses, which were fabricated to resemble those of natural teeth, can be preserved. One of the first steps to achieve the goal of fabricating precise fixed dental prostheses includes the accurate replication of mandibular movements. The procedure becomes complicated if the time required to replicate the mandibular movements is too long. If the device used to obtain the mandibular movements is too cumbersome, recording the movements may not be worthwhile. Therefore, incorporating a function to record mandibular movements within intraoral scanners can increase efficiency and simplify the procedure.

In the present study, the accuracy of the intraoral scanner’s ability to record the trajectory of movements was assessed in vitro using an articulator to exclude factors that may affect the quality of intraoral scan data, such as patient head movement and saliva or metal-based restorations. Instead of using commercially available typodonts, intraoral scan data from different participants were 3D-printed to evaluate their applicability for various arch widths, interarch relationships, and tooth arrangement circumstances.

Because the trajectory contains both length and direction factors, that is, its vector nature, it is difficult to define the accuracy of the trajectory between the two groups unless it is the same. For example, the trajectory length expressed in the two groups can be precisely the same; however, the direction can differ by 10°, and the start and end points of the trajectory can differ. However, how this accuracy should be expressed remains controversial. Moreover, because teeth have extremely complex and uneven structures, the trajectory may appear discontinuous, although it seems that it should appear on the same path. Therefore, in this proof-of-concept study, we expressed the accuracy of the trajectory in two parts: the difference in terms of the area of the trajectory and the difference in terms of the number of indented or marked trajectories. The analysis was further divided into tooth-type levels to elucidate potential differences according to the tooth type. Although the trajectories differed between the two groups, this may not necessarily indicate inaccuracies in the motion-tracking functionality of intraoral scanners. Further studies are needed to evaluate the clinical implications of these discrepancies, as actual mandibular movements in patients may differ from those simulated on an articulator. If the accuracy of the motion-tracking functionality of intraoral scanners is validated, these devices could serve as reliable tools for fabricating fixed dental prostheses.

This study had a few limitations. It was conducted in vitro, and only a single 3D-printable material and articulator were used. Additionally, because the study used an Acron-type articulator [15], its maxillary component had to be moved to represent the movements, which does not mirror the clinical situation. The values of the articulator were fixed in all 12 cases. How alterations in these factors would affect the outcomes requires further investigation. Finally, further exploration and discussion are warranted to define vectors with characteristic features. 

## 5. Conclusions

Intraoral scanners may not yet accurately capture motion movements with the current level of technology. However, these results should be interpreted with caution, as it is difficult to determine trajectory accuracy between the two groups unless their trajectories are identical.

## Figures and Tables

**Table 1:** The areas (mm^2^) of the trajectory of the two groups.

Tooth Type	ART Group	IOS Group	*p*-Value
Canine	458.5 (237.5–708.5)	247.5 (58.75–541.8)	0.002
First premolar	530.5 (366.3–933.8)	117.0 (0–299.8)	<0.001
Second premolar	401.5 (223.3–889.3)	87.0 (0–263.8)	<0.001
First molar	806.5 (570.5–1290.0)	173.5 (72.5–346.5)	<0.001
Second molar	803.0 (252.5–1162.0)	218.0 (111.5–284.8)	<0.001

## Data Availability

The data presented in this study are available upon request from the corresponding author.

## References

[1] Dawson P.E. (1996). A classification system for occlusions that relates maximal intercuspation to the position and condition of the temporomandibular joints. J. Prosthet. Dent..

[2] Warreth A., Ramadan M., Bajilan M.R., Ibieyou N., El-Swiah J., Elemam R.F. (2015). Fundamentals of occlusion and restorative dntistry. Part i: Basic principles. J. Iran. Dent. Assoc..

[3] Pokorny P.H., Wiens J.P., Litvak H. (2008). Occlusion for fixed prosthodontics: A historical perspective of the gnathological influence. J. Prosthet. Dent..

[4] Azer S.S., Kemper E. (2022). The patient-specific anatomical articulator. J. Prosthet. Dent..

[5] Carossa M., Cavagnetto D., Ceruti P., Mussano F., Carossa S. (2020). Individual mandibular movement registration and reproduction using an optoeletronic jaw movement analyzer and a dedicated robot: A dental technique. BMC Oral Health.

[6] Kordass B., Gärtner C., Söhnel A., Bisler A., Voss G., Bockholt U., Seipel S. (2002). The virtual articulator in dentistry: Concept and development. Dent. Clin. North Am..

[7] Röhrle O., Waddell J.N., Foster K.D., Saini H., Pullan A.J. (2009). Using a motion-capture system to record dynamic articulation for application in cad/cam software. J. Prosthodont..

[8] Coye R.B. (1977). A study of the variability of setting a fully adjustable gnathologic articulator to a pantographic tracing. J. Prosthet. Dent..

[9] Kwon J.H., Im S., Chang M., Kim J.E., Shim J.S. (2019). A digital approach to dynamic jaw tracking using a target tracking system and a structured-light three-dimensional scanner. J. Prosthodont. Res..

[10] Kinuta S., Wakabayashi K., Sohmura T., Kojima T., Mizumori T., Nakamura T., Takahashi J., Yatani H. (2005). Measurement of masticatory movement by a new jaw tracking system using a home digital camcorder. Dent. Mater. J..

[11] Farook T.H., Rashid F., Alam M.K., Dudley J. (2023). Variables influencing the device-dependent approaches in digitally analysing jaw movement-a systematic review. Clin. Oral Investig..

[12] Revilla-León M., Kois D.E., Zeitler J.M., Att W., Kois J.C. (2023). An overview of the digital occlusion technologies: Intraoral scanners, jaw tracking systems, and computerized occlusal analysis devices. J. Esthet. Restor. Dent..

[13] Baroudi K., Ibraheem S.N. (2015). Assessment of chair-side computer-aided design and computer-aided manufacturing restorations: A review of the literature. J. Int. Oral Health.

[14] Lee Y.C., Lee C., Shim J.S., Park J.M., Shin Y., Kim J.E., Lee K.W. (2020). Comparison between occlusal errors of single posterior crowns adjusted using patient specific motion or conventional methods. Appl. Sci..

[15] Beck H.O. (1959). A clinical evaluation of the arcon concept of articulation. J. Prosthet. Dent..

